# Supplementary material for: Causal mapping of psychological and occupational risk factors for suicidal ideation in psychiatric nurses using Bayesian networks: A multicenter cross-sectional study
Source: PLoS One. 2025 Sep 22;20(9):e0333018. doi: 10.1371/journal.pone.0333018 (PMC12453236; doi:10.1371/journal.pone.0333018)
Supplement: S1 Table — IQR, Interquartile range. (DOCX) [file pone.0333018.s001.docx]

**Supporting information**

**Causal mapping of psychological and occupational risk factors for suicidal ideation in psychiatric nurses using Bayesian networks: A multicenter cross-sectional study**

Min Wang et al.

**S1 Table. Descriptive statistics of continuous variables (overall sample)**

| Items | | Median | IQR | Min | Max |
| --- | --- | --- | --- | --- | --- |
| Burnout |  |  |  |  |  |
|  | Emotional exhaustion | 17 | 13 | 0 | 54 |
|  | Personal achievement | 17 | 17 | 0 | 48 |
|  | Depersonalization | 3 | 7 | 0 | 30 |
| Quality of work related-life |  |  |  |  |  |
|  | Working conditions | 23 | 5 | 6 | 30 |
|  | Stress at work | 20 | 5 | 6 | 30 |
|  | Control at work | 19 | 3 | 5 | 25 |
|  | Home-work interface | 8 | 1 | 2 | 10 |
|  | Employee engagement | 19 | 4 | 5 | 25 |
|  | General well-being | 19 | 3 | 5 | 25 |
|  | Job and career satisfaction | 15 | 2 | 5 | 20 |

IQR, Interquartile range.
