# Supplementary material for: Causal mapping of psychological and occupational risk factors for suicidal ideation in psychiatric nurses using Bayesian networks: A multicenter cross-sectional study
Source: PLoS One. 2025 Sep 22;20(9):e0333018. doi: 10.1371/journal.pone.0333018 (PMC12453236; doi:10.1371/journal.pone.0333018)
Supplement: S2 Table — OR, odds ratio; 95% CI, 95% confidence interval. (DOCX) [file pone.0333018.s002.docx]

**Supporting information**

**Causal mapping of psychological and occupational risk factors for suicidal ideation in psychiatric nurses using Bayesian networks: A multicenter cross-sectional study**

Min Wang et al.

**S2 Table. Factors associated with suicidal ideation identified by multivariate logistic regression**

| terms | estimate | OR (95% CI) | p value |
| --- | --- | --- | --- |
| Marry status | -0.257 | 0.774 (0.534, 1.12) | 0.174 |
| Income | 0.167 | 1.181 (0.848, 1.646) | 0.324 |
| Workplace force | 0.012 | 1.012 (0.717, 1.429) | 0.945 |
| Emotional exhaustion | 0.029 | 1.029 (1.007, 1.052) | 0.011 |
| Personal achievement | 0.029 | 1.029 (1.011, 1.048) | 0.002 |
| Depersonalization | 0.038 | 1.039 (1.004, 1.075) | 0.027 |
| Working conditions | 0.031 | 1.032 (0.956, 1.114) | 0.421 |
| Stress at work | -0.127 | 0.881 (0.83, 0.934) | <0.001 |
| Control at work | 0.026 | 1.026 (0.936, 1.125) | 0.579 |
| Home-work interface | 0.234 | 1.264 (1.079, 1.48) | 0.004 |
| Employee engagement | 0.027 | 1.027 (0.942, 1.12) | 0.541 |
| General well-being | -0.194 | 0.824 (0.757, 0.896) | <0.001 |
| Job and career satisfaction | -0.056 | 0.946 (0.839, 1.066) | 0.362 |

OR, odds ratio; 95% CI, 95% confidence interval.
