# Supplementary material for: Causal mapping of psychological and occupational risk factors for suicidal ideation in psychiatric nurses using Bayesian networks: A multicenter cross-sectional study
Source: PLoS One. 2025 Sep 22;20(9):e0333018. doi: 10.1371/journal.pone.0333018 (PMC12453236; doi:10.1371/journal.pone.0333018)
Supplement: Attachment — Submitted filename: Response to Reviewers.docx [file pone.0333018.s003.docx]

**Review Comments to the Author**

Please use the space provided to explain your answers to the questions above. You may also include additional comments for the author, including concerns about dual publication, research ethics, or publication ethics. (Please upload your review as an attachment if it exceeds 20,000 characters)

**Reviewer #1**: Recommendations for Manuscript ID PONE-D-25-36942 Title: “Causal mapping of psychological and occupational risk factors for suicidal ideation in psychiatric nurses using Bayesian networks: A multicenter cross-sectional study” for the Plos One Journal.

**General Comments**
From my point of view, it is a very interesting topic and simultaneously it seems that to the best of my knowledge is an empirical research aimed to present that psychiatric nurses represent a high-stress occupational group that experiences elevated levels of suicidal ideation (SI), which necessitates greater attention to their mental health. A total of 1,835 psychiatric nurses participated in the study, completing questionnaires that assessed depressive symptoms (PHQ-9), SI, quality of work-related life (QWL), and occupational burnout. Multivariate logistic regression and phenotypic network analyses, were conducted to identify factors associated with SI and explore the interactions and possible causal directions among depressive symptoms, burnout, QWL, and SI. The findings indicate that 11.33% of psychiatric nurses reported experiencing SI in the past two weeks. The multivariate logistic regression analysis identified emotional exhaustion, depersonalization, personal accomplishment, stress at work, general well-being, and home-work interface were significant as predictors of SI. In the symptom network, the top five symptoms directly connecting SI were psychomotor changes, guilt, sad mood, low energy, and appetite changes. Pathways were observed from sad mood, general well-being, and work-home interface to job and career satisfaction, as well as from sad mood, low energy to emotional exhaustion and SI. The study highlights the importance of targeting sad mood, psychomotor changes, low energy, emotional exhaustion, and work-related stress to reduce the risk of SI among psychiatric nurses.
**Response:** We sincerely thank the reviewer for the encouraging and positive comments on our work.

The paper contains the following sections: Introduction, Methods, Results, Discussion.
However, I find some recommendations:
1. The Manuscript needs careful English proofreading because there are some shortcomings. For instance, the article “the” is sometimes missing in front of nouns, the message in some paragraphs is not clear enough. It looks like the first part was written by one author with a greater command of the English language, and the rest of the paper was written by someone else. The numerous grammar errors made this a difficult paper to read. It was strange to see the authors refer to tables that were not submitted. I was unable to find any supplementary material to the submission, so I think this was truly omitted by the authors. Please read the manuscript carefully.

**Response:** We sincerely thank the reviewer for the valuable feedback. In the revised manuscript, we have thoroughly proofread the text for grammar, style, and clarity, corrected errors and rephrased ambiguous passages to increase readability and consistency.

With respect to the tables, the main text contains only one table, which has been retained. We have carefully rechecked the revised version to ensure that all the table references are accurate and consistent throughout the manuscript. Furthermore, as suggested, we have added two supplementary tables to the *Supporting Information*:

**“S1 Table. Descriptive statistics of continuous variables (overall sample)**

**S2 Table. Factors associated with suicidal ideation identified by multivariate logistic regression”**

2. The abstract must contain the main purpose of the paper, the research method used in the research and the main contributions.

**Response:** Following the recommendation, we have revised the *Abstract* to more clearly articulate the primary purpose of the study, the research methods employed, and the key contributions. The revised *Abstract* is presented below for your kind consideration:

“Psychiatric nurses represent a high-stress occupational group that experiences elevated levels of suicidal ideation (SI), emphasizing the need for focused mental health interventions. The main purpose of this study was to examine the prevalence of SI among psychiatric nurses and to identify the psychological and occupational factors associated with SI. A total of 1,835 psychiatric nurses completed questionnaires on depressive symptoms (PHQ-9), SI, quality of work-related life (QWL), and burnout. Multivariate logistic regression and phenotypic network analyses were conducted to identify factors associated with SI and the potential pathways linking depressive symptoms, burnout, and QWL to SI. The results indicated that 11.33% of the participants had SI in the past two weeks. Multivariate logistic regression revealed that emotional exhaustion, depersonalization, personal accomplishment, stress at work, general well-being, and the home-work interface were significant predictors of SI. Network analysis further revealed that psychomotor changes, guilt, sad mood, low energy, and appetite changes were the symptoms most directly associated with SI. In addition, sad mood, general well-being, and work-home interface were linked to job and career satisfaction, whereas sad mood and low energy were associated with emotional exhaustion and SI. These findings contribute valuable large-scale evidence on the mental health challenges faced by psychiatric nurses and highlight the importance of addressing mood disturbances, energy loss, and work-related stress in SI prevention efforts for this vulnerable group.” *(Lines 24-43)*

3. It would be very useful to add in the "Introduction" section the purpose, objectives and hypothesis of the research. I consider that a weak point of the paper is that the authors did not show the novelty of the paper compared to other works. That is why, I consider that the introduction should specify the novelty of the paper compared to other papers published in this area.

**Response:** We thank the reviewer for the insightful comment. Following this suggestion, we have revised the *Introduction* to include the purpose, objectives, and hypothesis of the study. The revised manuscript is as follows:

“The purpose of this study was to investigate the prevalence of SI among psychiatric nurses and to examine how depressive symptoms, burnout, and QWL interact to influence SI. We hypothesized that higher levels of burnout and depressive symptoms, coupled with lower QWL, would be associated with an increased risk of SI. To test these hypotheses, we applied multivariate regression to identify significant predictors and employed network analysis together with Bayesian causal models to capture the complex interrelationships among these factors. This integrative methodological approach extends previous research by moving beyond correlation-based analyses to infer potential causal pathways, thereby offering novel insights into central and bridge symptoms that may serve as effective intervention targets to reduce SI and improve the mental health of psychiatric nurses.” *(Lines 108-118)*

In addition, we have clarified the novelty of our research by emphasizing how our work integrates depressive symptoms, burnout, and QWL within a network and Bayesian framework, which differs from prior studies that examined these factors separately. The revised manuscript is as follows:

“Despite the recognized importance of burnout, QWL, and depressive symptoms in predicting suicide risk among healthcare professionals, most previous studies have examined these factors in isolation. This fragmented approach obscures the complex and dynamic interactions among them and limits the understanding of their combined impact on the SI. Addressing this gap requires an integrative approach that can capture both the complexity of symptom interactions and the potential causal pathways underlying them.

Network analysis provides such a framework. Unlike traditional methods that typically focus on linear relationships, network analysis visualizes and quantifies the relationships among how symptoms cluster and interact within a broader structure[18]. This approach has been particularly useful in mental health research, offering insights into the interplay of multiple symptoms and highlighting critical nodes—symptoms or variables that exert a disproportionate influence on the network and may represent potential targets for interventions[19, 20]. Complementing network analysis, directed acyclic graphs (DAGs) within a Bayesian network framework enable researchers to examine conditional dependencies and infer potential causal directions among variables [21]. By moving beyond correlation, DAGs help clarify which factors are more likely to directly shape the SI and which act through indirect pathways [22]. This capacity to map causal chains provides a richer understanding of how burnout, QWL, and depressive symptoms jointly contribute to SI among psychiatric nurses.” *(Lines 88-107)*

4. Literature review and Conclusion sections cannot be missing from the paper.

**Response:** We sincerely thank the reviewer for this valuable suggestion. In the revised manuscript, the relevant literature has been integrated into the *Introduction* section, and the main conclusions are summarized at the end of the *Discussion* section. Therefore, we believe that the essential elements of both the literature review and conclusion are now appropriately included while maintaining the overall structure required by the journal.

5. The research is well based on science, and the results are in agreement with the theoretical part.

**Response:** Thank you for the positive feedback. We are pleased that the study’s scientific basis and the consistency between theory and results were recognized.

6. I believe that the authors should also include other indicators from Descriptive Statistics.

**Response:** We sincerely thank the reviewer for this helpful suggestion.

1) In the revised manuscript, we have expanded the descriptive statistics to provide a more comprehensive presentation of the data. For continuous variables (burnout and QWL scores), in addition to reporting the means and standard deviations, we now also include the medians with interquartile ranges (IQRs) as well as the minimum and maximum values in the *Supporting Information* as follows:

**S1 Table. Descriptive statistics of continuous variables (overall sample)**

| Items | | Median | IQR | Min | Max |
| --- | --- | --- | --- | --- | --- |
| Burnout |  |  |  |  |  |
|  | Emotional exhaustion | 17 | 13 | 0 | 54 |
|  | Personal achievement | 17 | 17 | 0 | 48 |
|  | Depersonalization | 3 | 7 | 0 | 30 |
| Quality of work related-life |  |  |  |  |  |
|  | Working conditions | 23 | 5 | 6 | 30 |
|  | Stress at work | 20 | 5 | 6 | 30 |
|  | Control at work | 19 | 3 | 5 | 25 |
|  | Home-work interface | 8 | 1 | 2 | 10 |
|  | Employee engagement | 19 | 4 | 5 | 25 |
|  | General well-being | 19 | 3 | 5 | 25 |
|  | Job and career satisfaction | 15 | 2 | 5 | 20 |

IQR, Interquartile range.

2) With respect to clinical variables, we would like to clarify that only those listed in Table 1 were collected in the present study. We acknowledge the importance of including additional clinical indicators, and we will consider incorporating them in future research.

7. I think that the literature needs to be improved with other recent works, refers to rural tourism. That is why I recommend the authors to refer to other recent works indexed in Web of Science, Scopus, Emerald and Cambrige Journals. We suggest that the authors cite papers published in Web of Science Journals such as:
a. https://doi.org/10.3390/jrfm14070336
b. https://doi.org/10.1007/s13132-023-01565-6
c. <https://doi.org/10.3390/fractalfract8010032>

**Response:** We sincerely thank the reviewer for the kind recommendation and have carefully examined the suggested papers. While we find them valuable, their focus is not closely aligned with the specific scope of our study. Therefore, we have not included them in the current version. Nevertheless, we have strengthened the literature review by incorporating other recent and highly relevant works to better contextualize our research.

8. In conclusion, the article should be improved. It should also be enhanced with a review of the literature adequate to the subject and a broader interpretation and commentary of the research results.

**Response:** We sincerely thank the reviewer for this valuable comment. In the revised manuscript, we have refined the *Introduction* to better emphasize the novelty, expanded the *Discussion* to provide broader interpretation and significance, added a clearer account of the study’s limitations, and strengthened the *Conclusion* to highlight the contributions. We believe these revisions have improved the overall clarity and impact of the paper. The revised Discussion is as follows:

“Therefore, improving working conditions and promoting a better work-life balance could help alleviate emotional exhaustion while simultaneously enhancing career satisfaction and overall well-being. Consistent with the JD-R model, targeted interventions that support emotional health, such as structured mental health services, resilience-building strategies, and supervisory support, may yield meaningful improvements in both well-being and career satisfaction. Given the emotionally demanding nature of psychiatric nursing, even modest improvements in these areas are likely to yield not only increased professional fulfillment and quality of life but also broader organizational benefits, including reduced turnover intentions and improved quality of care[43-45].” *(Lines 331-340)*

“These findings underscore the need for dual-level interventions that recognize the continuum between burnout and depression [50]. At the organizational level, strategies aimed at mitigating exhaustion, such as adequate staffing, manageable workloads, and structured recovery opportunities, are critical, as exhaustion may represent an early point along the burnout–depression spectrum. At the individual level, timely screening and intervention for depressive symptoms, alongside support for energy restoration (e.g., sleep hygiene, fatigue management), can help prevent further progression along this continuum. Complementary resources that enhance personal accomplishment, including recognition programs, career development pathways, and peer support, may buffer against the detrimental effects of exhaustion, foster resilience, and potentially interrupt the trajectory from occupational stress to clinical depression.

In addition to confirming prior evidence, this research makes several original contributions. By applying network analysis and Bayesian DAG modeling, we move beyond simple correlations to uncover the causal structures that connect mood, energy, exhaustion, and suicidality. The identification of emotional exhaustion and low energy as “bridge symptoms” offers concrete leverage points for intervention, extending the JD-R model and Burnout–Depression Continuum Theory with new empirical evidence from psychiatric nurses.

Several limitations of this study should be acknowledged. First, this study focused on psychiatric nurses in Western China, which may restrict the generalizability of the findings to other healthcare populations or regions. Second, while the use of DAGs was appropriate for addressing our research objectives, the cross-sectional design of the study prevents us from establishing causality inference. Nevertheless, it enables us to uncover complex interrelationships among the variables, offering valuable insights. Third, participant recruitment through a self-developed digital platform may have introduced some selection bias, although the platform incorporated quality control measures to enhance data reliability. Fourth, reliance on self-reported measures raises concerns of reporting bias, especially for sensitive issues such as SI, where underreporting due to stigma or fear of repercussions cannot be excluded. Nevertheless, perceptions of emotions, stress, and SI are intrinsically subjective and thus most appropriately assessed through self-reports.

Future studies should employ longitudinal and cross-cultural designs to validate these pathways and examine the long-term effectiveness of both organizational and clinical interventions. Special attention should be given to minimizing response bias in self-reported suicidal ideation, possibly through indirect questioning or mixed-methods approaches. Strengthening the evidence base will not only advance scholarly understanding but also inform practical, evidence-driven strategies to protect psychiatric nurses' mental health and improve the sustainability and quality of mental health care systems.” *(Lines 356-394)*

**Reviewer #2:** The article does not specify the criteria for inclusiveness and exclusivity of the selection of respondents. A complete description of the respondents is not provided. The demographic and age distribution is not clear and therefore it is not clear whether the existing sample is sufficiently scientifically reliable and whether it corresponds to the general population.

**Response:** We sincerely thank the reviewer for this insightful comment. We have carefully revised the manuscript to address these concerns as follows:

**1) Inclusion and exclusion criteria**: We have explicitly added the inclusion and exclusion criteria to the *Methods* section as follows:

“The inclusion criteria for participants were registered nurses, aged 18-60 years who were currently employed in psychiatric institutions and had at least one year of experience in psychiatric clinical nursing. The participants were required to be aware of the study and to agree to participate voluntarily. Exclusion criteria: Nurses who had left for more than a month, student interns, those without a nursing license, or those who provided invalid responses (e.g., responses with an average time of less than one second per item, uniform answers across all items, or incomplete surveys) were excluded.” *(Lines 128-135)*

**2) Description of respondents**: In the revised manuscript, we have provided a more detailed description of the respondents in Table 1 and in the *Results* section, including their demographic characteristics and proportions.

“Among the 2,017 participants initially recruited, 1,835 (90.98%) respondents provided fully qualified data on all three scales (Table 1). The sample was predominantly female (88.7%) and younger than 30 years (52.2%). More than half (54.6%) had attained a bachelor’s degree or higher, and 68.1% were married. Most participants (73.6%) held junior or lower professional titles, whereas 29.7% reported a monthly income exceeding 5,000 RMB. Additionally, 39.5% had over 10 years of work experience, 46.3% engaged in night shifts more than four times per month, and 15.9% reported frequent overtime. Workplace violence was experienced by 28.6% of participants. Further details on burnout and the QWL can be found in S1 Table.” *(Lines 239-247)*

**3) Age distribution and representativeness**: We have clarified that the age range of the participants was 18–60 years. Considering the feasibility for participants during the survey, age and some other variables were collected as categorical rather than continuous data.

**Reviewer #3:** Suicidal ideation (SI) is more common among psychiatric nurses, a high-stress occupational group that requires more attention to their mental health. The study focuses on a significant topic. My observations are given below:

**Response:** We greatly appreciate the reviewer’s supportive comments.

(1) The study title is okay.

**Response:** We thank the reviewer for confirming that the study title is appropriate.

(2) The abstract needs revision. The abstract does a good job of summarizing the goal, methodology, and main conclusions of the study, but it is extremely technical and dense, which makes it difficult to read. While practical implications and novelty should be more prominently highlighted, excessive statistical detail could be trimmed. Increased clarity and reader engagement would result from a more focused emphasis on psychological significance.

**Response:** We sincerely appreciate your insightful comments on the abstract. In accordance with your suggestions, we have revised the manuscript to enhance clarity, reduce excessive technical detail, and better emphasize both the psychological significance and practical implications of our findings. The revised abstract is provided below:

“Psychiatric nurses represent a high-stress occupational group that experiences elevated levels of suicidal ideation (SI), emphasizing the need for focused mental health interventions. The main purpose of this study was to examine the prevalence of SI among psychiatric nurses and to identify the psychological and occupational factors associated with SI. A total of 1,835 psychiatric nurses completed questionnaires on depressive symptoms (PHQ-9), SI, quality of work-related life (QWL), and burnout. Multivariate logistic regression and phenotypic network analyses were conducted to identify factors associated with SI and the potential pathways linking depressive symptoms, burnout, and QWL to SI. The results indicated that 11.33% of the participants had SI in the past two weeks. Multivariate logistic regression revealed that emotional exhaustion, depersonalization, personal accomplishment, stress at work, general well-being, and the home-work interface were significant predictors of SI. Network analysis further revealed that psychomotor changes, guilt, sad mood, low energy, and appetite changes were the symptoms most directly associated with SI. In addition, sad mood, general well-being, and work-home interface were linked to job and career satisfaction, whereas sad mood and low energy were associated with emotional exhaustion and SI. These findings contribute valuable large-scale evidence on the mental health challenges faced by psychiatric nurses and highlight the importance of addressing mood disturbances, energy loss, and work-related stress in SI prevention efforts for this vulnerable group.” *(Lines 24-43)*

(3) The introduction also needs minor revision. A thorough and thoroughly referenced background is provided in the introduction, which successfully places the study in the context of psychiatric nurses' risk of suicide. Nevertheless, it is excessively long and has repetitions that mask the main research void. It feels sudden and could be more smoothly integrated to go from general suicide risk to methodological options (network analysis, DAGs). Readability and theoretical coherence would be enhanced by more precise articulation of hypotheses and a greater emphasis on the psychological processes that connect burnout, QWL, and suicidal thoughts.

Response: We thank the reviewer for this helpful suggestion.

1) The Introduction has been shortened to remove repetitions and improve readability.

“Suicide remains a significant global concern, causing over 817,000 deaths annually and accounting for 1.49% of all deaths worldwide[1]. Among healthcare professionals, psychiatric nurses are particularly vulnerable, and have significantly higher suicide rates than the general population does[2, 3]. Elevated levels of suicidal ideation (SI) in this group are largely attributed to the intense emotional demands and chronic job-related stress inherent in their profession[4, 5], highlighting the urgent need for targeted mental health support and interventions.” *(Lines 48-54)*

2) While making the transition to the methodological rationale (network analysis and DAGs) smoother.

“Despite the recognized importance of burnout, QWL, and depressive symptoms in predicting suicide risk among healthcare professionals, most previous studies have examined these factors in isolation. This fragmented approach obscures the complex and dynamic interactions among them and limits the understanding of their combined impact on the SI. Addressing this gap requires an integrative approach that can capture both the complexity of symptom interactions and the potential causal pathways underlying them.

Network analysis provides such a framework. Unlike traditional methods that typically focus on linear relationships, network analysis visualizes and quantifies the relationships among how symptoms cluster and interact within a broader structure[18]. This approach has been particularly useful in mental health research, offering insights into the interplay of multiple symptoms and highlighting critical nodes—symptoms or variables that exert a disproportionate influence on the network and may represent potential targets for interventions[19, 20]. Complementing network analysis, directed acyclic graphs (DAGs) within a Bayesian network framework enable researchers to examine conditional dependencies and infer potential causal directions among variables [21]. By moving beyond correlation, DAGs help clarify which factors are more likely to directly shape the SI and which act through indirect pathways [22]. This capacity to map causal chains provides a richer understanding of how burnout, QWL, and depressive symptoms jointly contribute to SI among psychiatric nurses.” *(Lines 88-107)*

3) We also clarified the hypotheses and strengthened the link between burnout, QWL, depressive symptoms, and suicidal ideation.

“We hypothesized that higher levels of burnout and depressive symptoms, coupled with lower QWL, would be associated with an increased risk of SI.” *(Lines 110-111)*

(4) The method again needs minor amendments. The methods section is thorough, shows exacting statistical and measurement procedures, and reports on the reliability of every instrument. However, generalizability may be limited due to the cross-sectional design, which limits causal inference, and the lack of discussion regarding the rationale behind the exclusive selection of psychiatric nurses from Sichuan Province. Although DAG and network analyses are novel, assumptions like multivariate normality need more convincing evidence. Deeper thought should be given to the selection bias and data validity issues brought up by the hiring process using a self-developed digital platform.

**Response:** We thank the reviewer for these thoughtful suggestions.

1) In the revised *Methods*, we outline the quality-control steps embedded in our self-developed digital platform as follows:

“Exclusion criteria: Nurses who had left for more than a month, student interns, those without a nursing license, or those who provided invalid responses (e.g., responses with an average time of less than one second per item, uniform answers across all items, or incomplete surveys) were excluded.” *(Lines 131-135)*

2) The revised *Limitations* regarding the focus on psychiatric nurses in Sichuan Province are as follows:

“First, this study focused on psychiatric nurses in Western China, which may restrict the generalizability of the findings to other healthcare populations or regions.” *(Lines 374-376)*

3) We also note that, while GGMs assume multivariate normality, we examined distributional properties and applied regularized and bootstrap-based procedures to improve robustness.

“To enhance the robustness and interpretability of the network, we additionally calculated the frequency of each edge across 10,000 bootstrap samples in the Bayesian network structures.” *(Lines 231-233)*

4) In addition, the cross-sectional design and external validity concerns are explicitly acknowledged in the revised Limitations section as follows:

“Second, while the use of DAGs was appropriate for addressing our research objectives, the cross-sectional design of the study prevents us from establishing causality inference. Nevertheless, it enables us to uncover complex interrelationships among the variables, offering valuable insights.” *(Lines 376-379)*

(5) The results need modifications. With its thorough analysis and integration of several statistical techniques, the results section provides deep insights into the connections between SI, burnout, QWL, and depressive symptoms. On the other hand, overly detailed figures and edge weights make it difficult to read and hide important points. Without a clear synthesis, some results are replicated in network and regression analyses. Interpretation would be strengthened by a stronger focus on the practical implications of effect sizes and a more distinct differentiation between statistical significance and clinical relevance. Simplifying would increase clarity without sacrificing the depth of the science.

**Response:** We greatly appreciate the reviewer’s helpful feedback. In line with these suggestions, we have revised the *Results* section to improve clarity, reduce redundancy, and strengthen the focus on practical implications and clinical relevance. We revised the *Results* section as follows:

“**Factors associated with SI: results of multivariate logistic regression analyses**

The multivariate logistic regression model (Fig 1, S2 Table) confirmed that several burnout and AWL variables were independently associated with SI. Emotional exhaustion (OR = 1.029, 95% CI: 1.007-1.05, *p* = 0.011), depersonalization (OR = 1.039, 95% CI: 1.004-1.075, *p* = 0.027), and personal accomplishment (OR = 1.029, 95% CI:1.011-1.048, *p* = 0.002) were identified as significant risk factors. In contrast, lower stress at work (OR = 0.881, 95% CI:0.83-0.934, *p* < 0.001), and greater general well-being (OR = 0.824, 95% CI:0.757-0.896, *p* <0 .001) were protective. Additionally, home-work interfaces (OR = 1.264, 95% CI:1.079-1.480, *p* = 0.004) played a notable role. While the effect sizes were generally modest, their cumulative impact suggests that burnout and poor QWL, when occurring simultaneously, substantially elevate the risk of SI. This highlights the importance of workplace interventions that target multiple dimensions rather than focusing on isolated factors.

**Phenotype network of burnout, QWL, depressive symptoms, and SI**

The network model incorporated 19 psychological phenotypes, yielding 171 potential edges, of which 106 (62.0%) were retained after regularization (Fig 2A-C). Among these, 74 represented positive associations and 32 represented negative associations. The strongest direct edges connecting SI were with psychomotor changes, guilt, sad mood, energy, and appetite.

Centrality analysis revealed that low energy and emotional exhaustion as the most influential nodes in terms of strength, whereas sad mood was second-ranked in terms of expected influence (Fig 2D). Bridge analyses further revealed emotional exhaustion and low energy as critical connections linking occupational burnout and depressive symptoms (Fig 2E). Taken together, these results indicate that fatigue-related and affective symptoms not only cluster around SI but also act as bridges across symptom domains, underscoring their potential as key intervention targets.

**Bayesian network analysis**

Bayesian network analysis provided complementary insights into the directionality of associations (Fig 3). General well-being influences several QWL domains, including employee engagement, work-related stress, control at work, and the home-work interface, while also indirectly affecting burnout and depressive symptoms. Job and career satisfaction emerged as the most influential aspects of QWL, shaped by both occupational and emotional factors such as, control at work, working conditions, sleep disturbances and concentration issues. Among the depressive symptoms, sad mood was the most impactful and was associated with psychomotor changes, anhedonia, sleep disturbances, feelings of guilt, low energy, concentration difficulties, SI, depersonalization, emotional exhaustion, general well-being, and work-related stress. Psychomotor changes are driven primarily by other depressive symptoms but are also connected to depersonalization, highlighting the overlap between the affective and occupational domains. Finally, SI was most strongly predicted by sad mood, low energy, guilt, and psychomotor changes, which is consistent with the network findings. These patterns suggest that addressing both workplace-related and depressive factors, particularly those involving energy and mood regulation, may provide the most effective strategy for reducing SI risk among psychiatric nurses.” *(Lines 255-300)*

(6) The discussion needs minor improvements. The discussion is comprehensive, clearly defining two main pathways and connecting findings to pertinent theories and earlier research. Integration of the Burnout-Depression Continuum Theory and the JD-R model is one of its strengths. Nevertheless, some passages are repetitive and excessively detailed, which lessens their impact. Its applicability would be increased by placing more focus on original contributions and useful intervention techniques. Although the limitations section is appropriate, it could more thoroughly address the possibility of response bias in delicate subjects like SI. All in all, a good but unduly long conversation.

**Response:** We thank the reviewer for the positive evaluation of our discussion and valuable suggestions for improvement. We have revised the *Discussion* to reduce repetition and enhance clarity, placed greater emphasis on the study’s original contributions and practical implications, and expanded the *Limitations* to more explicitly address potential response bias. The revised *Discussion* and *Limitations are* as follows:

“Therefore, improving working conditions and promoting a better work-life balance could help alleviate emotional exhaustion while simultaneously enhancing career satisfaction and overall well-being. Consistent with the JD-R model, targeted interventions that support emotional health, such as structured mental health services, resilience-building strategies, and supervisory support, may yield meaningful improvements in both well-being and career satisfaction. Given the emotionally demanding nature of psychiatric nursing, even modest improvements in these areas are likely to yield not only increased professional fulfillment and quality of life but also broader organizational benefits, including reduced turnover intentions and improved quality of care[43-45].” *(Lines 331-340)*

“These findings underscore the need for dual-level interventions that recognize the continuum between burnout and depression [50]. At the organizational level, strategies aimed at mitigating exhaustion, such as adequate staffing, manageable workloads, and structured recovery opportunities, are critical, as exhaustion may represent an early point along the burnout–depression spectrum. At the individual level, timely screening and intervention for depressive symptoms, alongside support for energy restoration (e.g., sleep hygiene, fatigue management), can help prevent further progression along this continuum. Complementary resources that enhance personal accomplishment, including recognition programs, career development pathways, and peer support, may buffer against the detrimental effects of exhaustion, foster resilience, and potentially interrupt the trajectory from occupational stress to clinical depression.

In addition to confirming prior evidence, this research makes several original contributions. By applying network analysis and Bayesian DAG modeling, we move beyond simple correlations to uncover the causal structures that connect mood, energy, exhaustion, and suicidality. The identification of emotional exhaustion and low energy as “bridge symptoms” offers concrete leverage points for intervention, extending the JD-R model and Burnout–Depression Continuum Theory with new empirical evidence from psychiatric nurses.” *(Lines 356-373)*

“Third, participant recruitment through a self-developed digital platform may have introduced some selection bias, although the platform incorporated quality control measures to enhance data reliability. Fourth, reliance on self-reported measures raises concerns of reporting bias, especially for sensitive issues such as SI, where underreporting due to stigma or fear of repercussions cannot be excluded. Nevertheless, perceptions of emotions, stress, and SI are intrinsically subjective and thus most appropriately assessed through self-reports.” *(Lines 380-386)*
